# Identification of angiogenesis-related subtypes, the development of prognostic models, and the landscape of tumor microenvironment infiltration in colorectal cancer

**DOI:** 10.3389/fphar.2023.1103547

**Published:** 2023-02-22

**Authors:** Chen Zhang, Tao Liu, Zhennan Yun, Bin Liang, Xue Li, Jiantao Zhang

**Affiliations:** Colorectal and Anal Surgery Department, General Surgery Center, First Hospital of Jilin University, Changchun, Jilin, China

**Keywords:** angiogenesis, colorectal cancer, tumor microenvironment, prognosis, immunotherapy

## Abstract

**Background:** Angiogenesis is one of the most prominent markers of cancer progression and contributes to tumor metastasis and prognosis. Anti-angiogenic drugs have proven effective in treating metastatic colorectal cancer. However, there is some uncertainty regarding the potential role of angiogenesis-related genes in the tumor microenvironment.

**Methods:** We analyzed 1,214 colorectal cancer samples to identify alterations in angiogenesis-related genes (ARGs), and then correlated angiogenesis with clinical features, prognosis, and TME. The ARGs expression profiles in colorectal cancer were analyzed using three computational methods (CIBERSORT, ssGSEA, and MCPcounter) and provided a systematic immune landscape. Patients with CRC were classified into two subtypes based on consensus clustering analysis of angiogenesis-related genes. The revealed differentially expressed genes between the two subtypes were used to create and validate ARGscore prognostic models. In addition, we collected eight colorectal cancer patient specimens and performed RT-qPCR to validate the signature gene expression.

**Results:** We assessed the expression patterns of ARGs in colorectal cancer. We identified two molecular subtypes and confirmed that the expression of ARGs was associated with prognosis and TME characteristics. Based on differentially expressed genes between subtypes, we constructed ARGscore and evaluated their predictive power for the survival of colorectal cancer patients. We also developed an accurate nomogram to make the ARGscore more clinically useful. In addition, ARGscore was significantly correlated with microsatellite instability, cancer stem cells, and chemotherapeutic drug sensitivity. Patients with ARGscore-low characterized by immune activation and microsatellite instability high had a better prognosis.

**Conclusion:** ARGs expression influenced the prognosis, clinicopathological features, and tumor stromal immune microenvironment in colorectal cancer. We developed a new risk model, ARGscore, for the treatment and prognosis of CRC patients and validated its promising predictive power. These findings will enable us to understand colorectal cancer better, assess prognoses, and develop more effective treatment options.

## Introduction

Angiogenesis, the production of new capillaries from blood vessels, is a critical process in several physiological and pathological processes, including organic growth and development, wound healing, tumor growth, metastasis, etc. [(Kuczynski et al., 2019; Jiang et al., 2020)]. The process of tumor angiogenesis is highly complex and dynamic, involving steps such as the migration of endothelial cells, degradation of the vascular endothelial matrix, the proliferation of endothelial cells, and the formation of new basement membranes [(Folkman, 1971; Weidner et al., 1991)]. Large endothelial gaps, an incomplete vascular matrix, significant vascular permeability, and structural and functional defects are all signs of tumor neovascularization (Parmar and Apte, 2021). The growth and progression of many malignancies, including colorectal cancer, depend on angiogenesis [(Viallard and Larrivee, 2017; Chen et al., 2021)].

The morbidity and mortality rates in colorectal cancer (CRC) are third and second highest in the world, respectively (Sung et al., 2021). CRC development and progression are strongly influenced by angiogenesis. Pro-tumor vascular growth factors so far discovered include vascular endothelial growth factor (VEGF) and its receptor vascular endothelial growth factor receptor (VEGFR), which stimulate tumor vascular endothelial cell growth and migration, increase tumor neovascularization permeability, and play an essential role in the angiogenesis process (Ferrara, 2004; Jiang and Liu, 2009; Claesson-Welsh and Welsh, 2013; Apte et al., 2019). VEGF expression is associated with a worse prognosis in colorectal cancer. VEGFR also correlated with colorectal Duke-stage, tumor grade, and lymph node involvement (Rmali et al., 2007).

The extracellular matrix, fibroblasts, endothelial cells, cytokines, and chemokines that these cells release, as well as the tumor tissue that has been surrounded or infiltrated by immune and inflammatory cells, make up the tumor microenvironment (TME) (Pottier et al., 2015). TME is frequently thought to be connected to the growth and spread of tumors. By releasing cell signaling molecules, malignant cells promote tumor angiogenesis and immune tolerance by communicating with surrounding cells [(Runa et al., 2017; Jin and Jin, 2020)]. Mesenchymal cells and fibroblasts in tumor tissue secrete fibroblast growth factor (FGF), chemokine (C-X-C Motif) ligand 12 (CXCL12), and matrix metallopeptidase 2 (MMP2) to enhance tumor cell growth, invasion, and metastasis [(Hanahan and Coussens, 2012; Wang et al., 2017)]. Currently, individual ARGs are being studied in most studies concerning colorectal cancer progression and prognosis, and anti-angiogenic therapy has been used as an important tool for oncology treatment [(El-Kenawi and El-Remessy, 2013; Ramjiawan et al., 2017)]. Therefore, it is important to understand the characteristics of multiple ARG-mediated TME to gain a deeper understanding of colorectal carcinogenesis and predict the response to immunotherapy.

In this study, ARG expression profiles of colorectal cancer were analyzed using multiple bioinformatics approaches and provided a systematic immune landscape. Based on ARG expression, CRC patients were classified into two subtypes, and differentially expressed genes (DEGs) were identified between subtypes. In addition, we developed the ARGscore to predict overall survival (OS) and immunotherapy response in CRC patients and validated its accuracy. We also investigated the potential association between angiogenesis, the tumor immune microenvironment, the prognosis of CRC patients, and response to immunotherapy.

## Materials and methods

### Data processing

The workflow is shown in Supplementary Figure S1 for our study. Gene expression and clinical characteristics of CRC samples were retrieved from GEO databases (https://www.ncbi.nlm.nih.gov/geo/) and TCGA (https://portal.gdc.cancer.gov/). Two GEO datasets (GSE39582 and GSE17536) and the TCGA-COAD/READ cohort were included for further analysis. Further evaluation of patients without survival information was not conducted. As part of the data processing, we adjusted the background and normalized the quantiles of the data. For the dataset in TCGA, we converted the downloaded Fragments Per Kilobase Million (FPKM) value to the Transcripts per million (TPM) value, which was thought to be the same as the microarrays (Conesa et al., 2016). The batch effect for non-biotechnology bias was corrected by the “battle” algorithm of the SVA software, and all datasets were combined. Supplementary Table S1 summarized specific information for all eligible patients with CRC. Data on somatic mutations were downloaded from the TCGA. The UCSC Xena database (https://xena.ucsc.edu/) was used to download copy number variation (CNV) for the TCGA cohort.

### Consensus clustering analysis of angiogenesis-related genes

We retrieved the 36 angiogenesis-related genes (ARGs) from MSigDB (Hallmark gene sets). ConsensuClusterPlus was utilized to perform an unsupervised clustering analysis to identify different angiogenesis subtypes according to the expression of 36 ARGs. Principal component analysis (PCA) was performed to show the distribution difference of angiogenesis subtypes. Functional annotation and gene set variance analysis (GSVA) to discover the discrepancy in biological processes among subtypes. GSVA was performed on the gene set derived from the MSigDB database (C2. Cp.ke.v7.2), and adjusted *p* < 0.05 was statistically significant. Based on Kaplan-Meier curves created from the “survival” and “survival” R packages, we assessed the differences in OS between the two clusters.

### Correlation of distinct subtypes with TME

To estimate the proportion of immune cells infiltrating CRC samples, we used the CIBERSORT algorithm, in which 22 immune cell populations were distinguished using cell-specific gene signatures (Newman et al., 2015). The single sample gene set enrichment analysis (ssGSEA) was also used to determine the level of immune cell infiltration in the TME [(Barbie et al., 2009; Charoentong et al., 2017)]. Next, we used the microenvironmental cell population algorithm (MCPcounter) to quantify the proportion of fibroblasts and endothelial cells (Becht et al., 2016). The ESTIMATION algorithm is also used to assess the immune and stromal score of each patient.

### Construction of the ARGscore

To determine differentially expressed genes (DEGs) between different subtypes, we used the limma package of R with a criterion of |log2FC| ≥ 1, and the adjusted *p*-value was <0.01. We were using the Clusterprofiler package to explore further the gene function and enrichment pathway associated with DEGs. Then, DEGs associated with survival were identified using univariate Cox regression analysis. Patients were divided into different gene subtypes for in-depth analysis based on prognosis-related ARGs. With the Lasso Cox regression algorithm and the “glmnet” R package, CRC patients were divided randomly into training and test sets according to prognostic genes. Using multivariate Cox analysis, candidate genes were screened to create ARGscore that quantified each tumor pattern in the training set. The ARGscore was calculated by multiplying the gene expression values by their risk coefficients. Kaplan-Meier survival analysis based on the median risk score was performed on the train set. We divided the test and all sets into ARGscore-low and ARGscore-high subgroups, and each subgroup underwent Kaplan-Meier survival analysis and ROC curve generation.

### RNA extraction in tissue samples and RT-qPCR

Normal and colorectal tumor tissue was collected from eight patients with colorectal cancer at the First Hospital of Jilin University. We stored the samples at −80°C until use. We extracted total RNA from tissues with TRIzol (GenStar, China), and its purity and concentration were determined. We reversed transcribed RNA into cDNA using Uni RT&qPCR kit (transgen, China) and performed RT-qPCR (real-time quantitative polymerase chain reaction) on the instrument (ABI QuantStudio 3, United States). We used GAPDH as an internal reference, and calculated relative gene expression levels with 2^−ΔΔCT^, visualizing data with Graphpad 8.0. A detailed list of primer sequences used for RT-qPCR is provided in Supplementary Table S2. The experiments were undertaken with the understanding and written consent of each subject. The study methodologies conformed to the standards set by the Declaration of Helsinki. The study methodologies were approved by the First Hospital of Jilin University ethics committee.

### Clinical correlation analysis of ARGscore

The correlation between ARGscore and clinical characteristics was investigated. Using univariate and multivariate analyses, we assessed whether the ARGscore is separate from other clinical factors as a prognostic indicator. We quantified 22 infiltrating immune cells using CIBERSORT and compared their levels between subgroups. Moreover, immune checkpoint expression levels were compared between distinct groups. Additionally, we examined the correlation between the ARGscore, microsatellite instability (MSI), and cancer stem cell (CSC). The waterfall function of the “mafTools” package was used to present mutations in the TCGA-COAD/READ cohort in order to differentiate somatic mutations from ARGscore-low and ARGscore-high subgroups. We calculated the IC_50_ of the used chemotherapeutic agents for CRC using the pRophetic package to explore the differences in efficacy between the two subgroups.

### Statistical analysis

Univariate survival analysis with Kaplan-Meier and log-rank tests. Multifactor survival analysis was conducted using COX regression models. By calculating the area under the curve using the pROC software package, we were able to assess the specificity and sensitivity of ARGscore. The continuous variables were compared using independent t-tests. Categorical data were tested with chi-square tests. Statistical significance was defined as a two-sided *p*-value < 0.05. All data processing was done in R4.0.4 software.

## Result

### Genetic variation of ARGs in colorectal cancer

**FIGURE 1:**
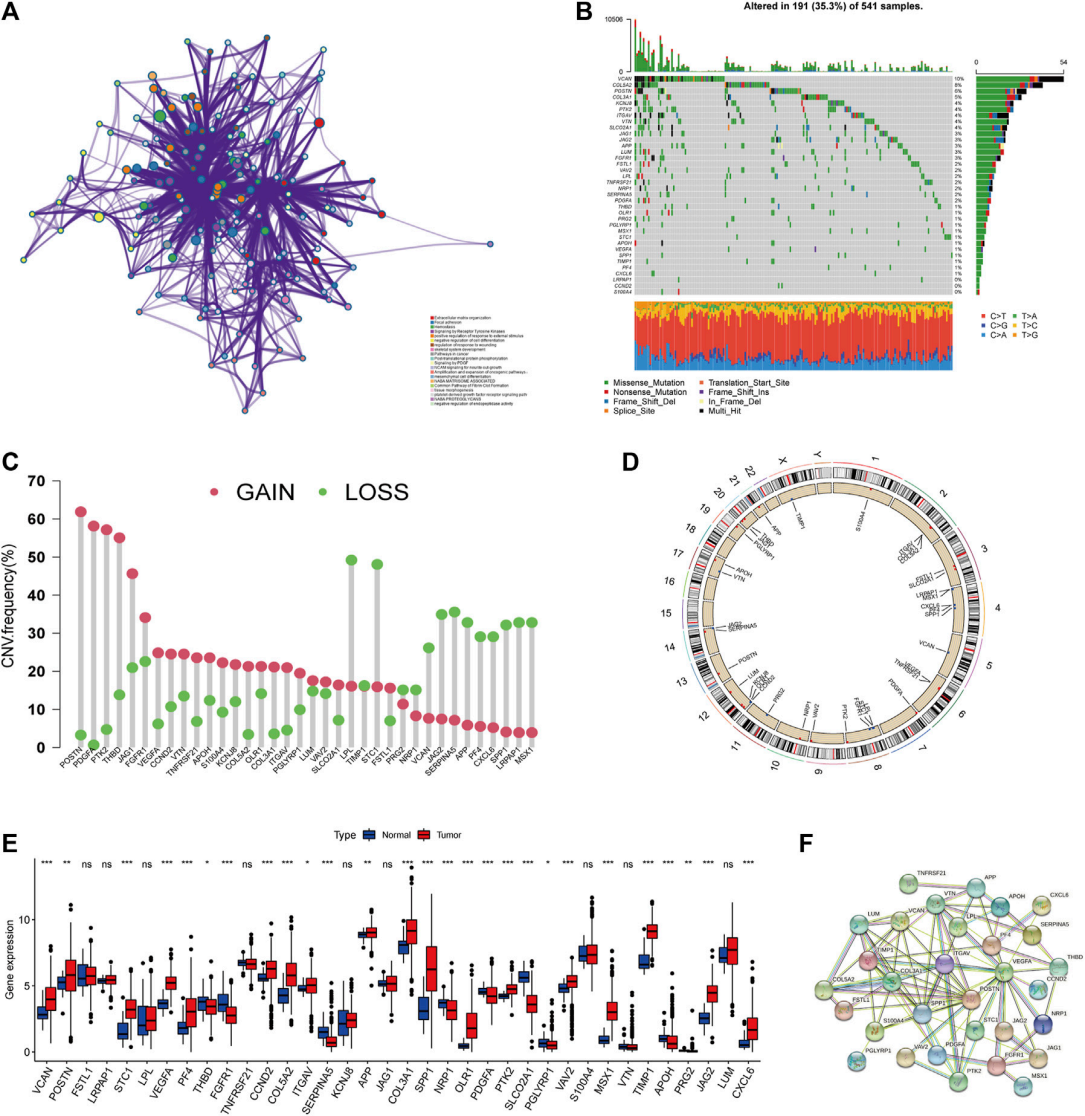
Genetic landscape of ARGs in colorectal cancer. **(A)** Metascape enrichment network visualization reveals biological functions and pathways of ARGs. Cluster annotations were shown in the color code. **(B)** Mutation frequency of ARGs in TCGA COAD/READ cohort. Of the 541 CRC patients, 191 had angiogenesis-related gene alterations with a frequency of 35.3%, consisting mainly of missense mutations, multi-hit, and nonsense mutations. The numbers on the right indicate the frequency of mutations in each gene. Each column represents an individual patient. **(C)** Frequency of CNV gain and loss in ARGs. The column represented the alteration frequency. The gain frequency, red dot; The loss frequency, green dot. **(D)** The location of CNV alteration of ARG on chromosomes. **(E)** Expression of 36 ARGs in normal and CRC tissues. The asterisks represented the statistical *p*-value (*p < 0.05; **p < 0.01; ***p < 0.001). **(F)** The major PPI network in ARGs is from the STRING database.

We retrieved and investigated 36 ARGs in our study (Supplementary Table S3). Metascape and GO analysis of 36 ARGs were performed, and Figure 1A and Supplementary Figure S2A summarized the significantly enhanced biological processes. For studying the genomic features of ARGs in CRC, the analysis of the somatic mutation frequency of the 36 ARGs in CRC indicated that 191(35.3%) of the 541 samples had mutations. In CRC samples, VACN and COL5A2 had the highest mutation frequencies (10% and 8%), while LRPAP1, CCND2, and S100A4 did not exhibit any mutations (Figure 1B). Further investigation revealed significant relationships between the expression of SERPINA5, TNFRSF21, PTK2 and VCAN mutations (Supplementary Figure S2B). Next, we analyzed somatic copy number alterations in ARGs. We found that CNV alterations were prevalent in 36 ARGs, and most showed extensive CNV amplification, while LPL, STC1, SERPINA5, and JAG2 showed reduced CNV (Figure 1C). The chromosome positions of CNV-altered ARGs are shown in Figure 1D. We subsequently analyzed the mRNA expression levels of ARGs in colorectal cancer and normal tissues to determine whether the aforementioned genetic variations impact the expression of ARGs in CRC patients. We discovered that most ARGs were highly expressed in tumor tissues (Figure 1E). The expression of CNV-deficient ARGs, including SERPINA5, was lower in tumor tissues than in normal colorectal tissues, indicating that CNV might regulate the expression of ARGs. CNV-gained ARGs, including PDGFA, were profoundly downregulated in tumor tissues, whereas CNV-amplified and deletion ARGs were not significantly differentially expressed. These also indicated that mRNA expression is not only regulated by CNV. In addition, PPI analysis on the STRING showed major ARGs interaction (Figure 1F). As a result of the above analyses, significant differences were found in gene and expression profiles of ARGs in colorectal cancer and normal samples, supported their role in colorectal cancer.

### Identification of ARGs-mediated colorectal cancer subtype

**FIGURE 2:**
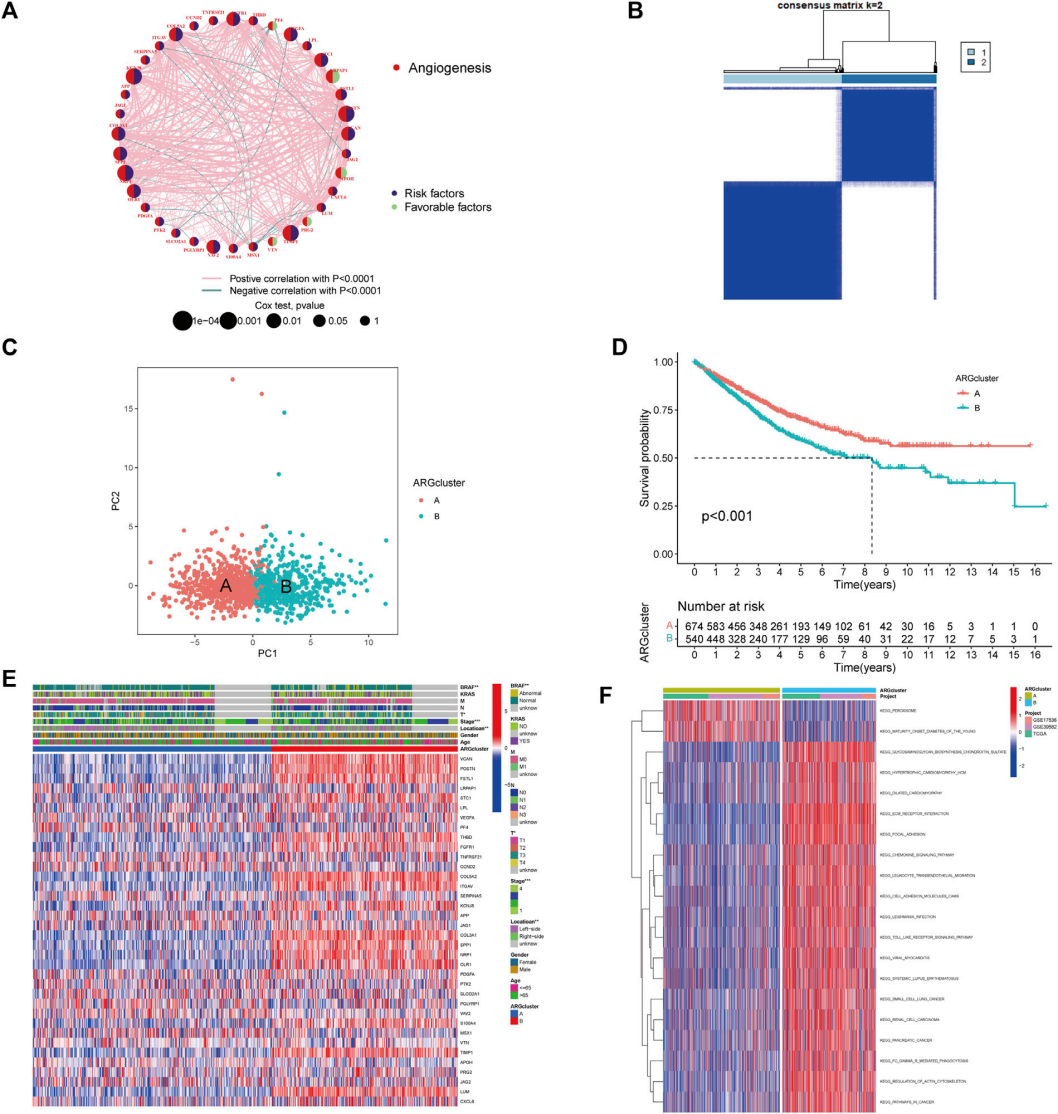
Identification of angiogenesis subtypes and relevant biological pathways. **(A)** The Interaction network between ARGs in CRC. The circles represented the different ARGs and the circle size represented the effect of each gene on prognosis, with values ranging from *p* < 0.0001, *p* < 0.001, *p* < 0.01, *p* < 0.05, and *p* < 1, respectively, by the Log-rank test. purple in the circle, prognostic risk factors; green in the circle, prognostic protective factors. The lines connecting genes indicated their interactions, and the thickness indicated the strength of the association between regulators. Pink color indicated positive association, green color indicated negative association. **(B)** Heatmap of consensus matrix defining two clusters. **(C)** The PCA analysis revealed significant differences between subtypes. **(D)** Kaplan-Meier curves of OS for CRC patients in the cohort (Log-rank test, *p* < 0.001). **(E)** Differences in clinicopathological characteristics and expression levels of ARGs in two subtypes. **(F)** Differences in biological pathways among subtypes as determined by the GSVA.

For further analysis, 1,214 patients from colorectal cancer datasets were collected (TCGA-COAD/READ, GSE39582, and GSE17536). Based on univariate COX regression analysis and Kaplan-Meier analysis, 36 ARGs were evaluated for their prognostic value in CRC patients (Supplementary Figure S2C; Supplementary Table S4). LRPAP1 and APOH could be considered protective factors, while VEGFA and FGFR1 could be considered risk factors. Figure 2A and Supplementary Table S5 illustrated a complete network of the ARG interaction, the connection of regulators, and their prognostic value in CRC patients. Subsequently, CRC patients were unsupervised clustered, and two subtypes were identified (Figure 2B; Supplementary Figure S3): subtype A (*n* = 674) and subtype B (*n* = 540). Based on PCA analysis, these two subtypes of ARGs exhibited significantly different transcriptional profiles (Figure 2C). Kaplan-Meier curves illustrated that subtype A exhibited a significant survival advantage (Figure 2D). Furthermore, we compared the gene expression and clinicopathological characteristics of the two subtypes (Figure 2E). The subtype A was associated with left-side (*p* < 0.01), lower stage (*p* < 0.001), and without BRAF mutation (*p* < 0.01) compared with subtype B.

### Characterization of TME in distinct subtypes

**FIGURE 3:**
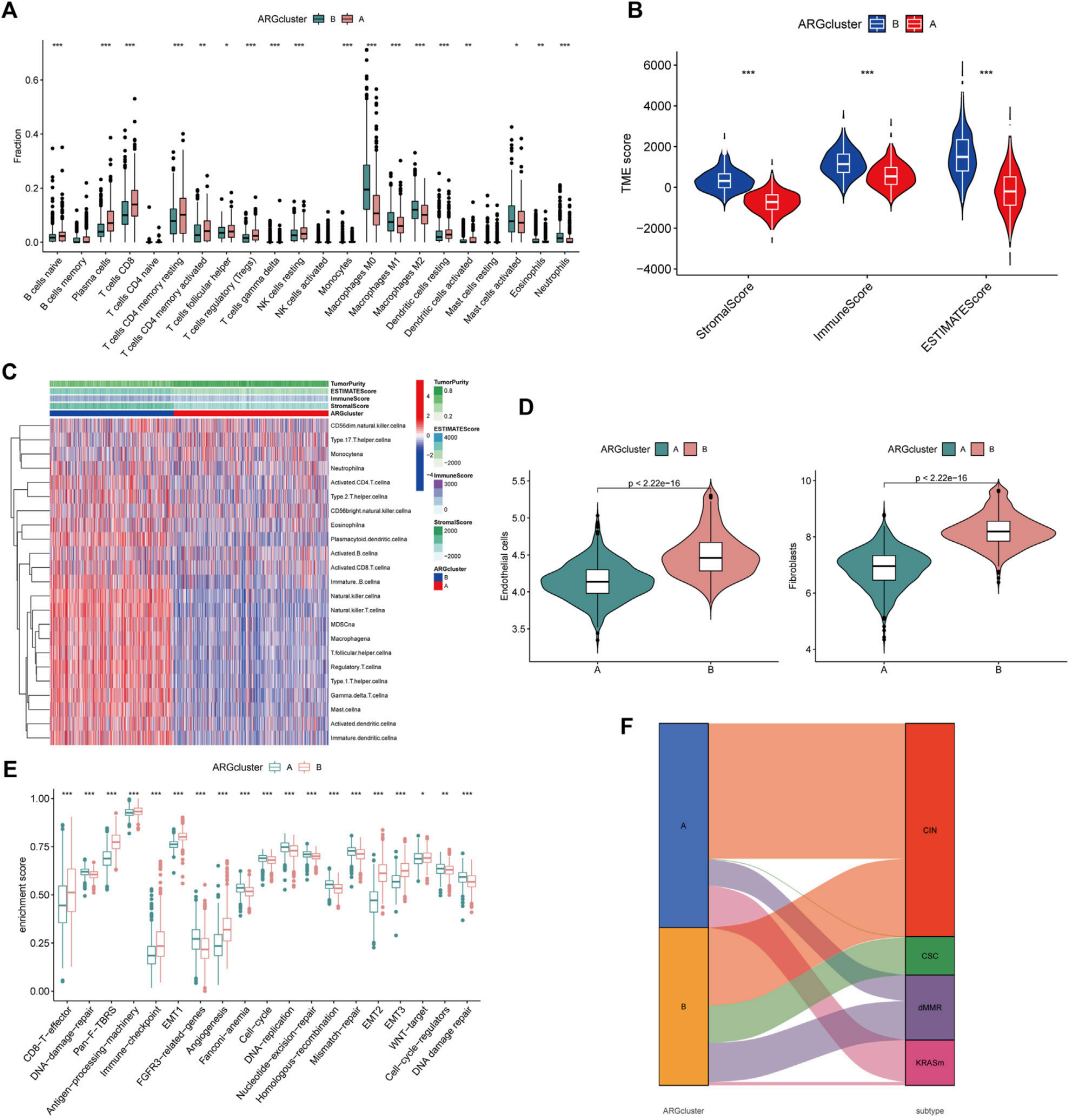
Differential TME characteristics **(A)** The proportion of immune cell infiltration in the two angiogenesis clusters was calculated using the CIBERSORT algorithm. The upper and lower ends of the boxes represented interquartile range of values. The lines in the boxes represented median value, and black dots showed outliers. The asterisks represented the statistical *p*-value (*p < 0.05; **p < 0.01; ***p < 0.001). **(B)** Relationship between TME score and subtypes (*p < 0.05; **p < 0.01; ***p < 0.001). **(C)** Heatmap was constructed using ssGSEA to visualize and compare the relative abundance, mesenchymal score, immune score, and tumor purity of 23 immune infiltrating cell subpopulations under different clusters. **(D)** Differences in endothelial cells and fibroblasts in different clusters were evaluated using the MCPcounter algorithm. The thick line represented the median value. The bottom and top of the boxes represented interquartile range of values. The whiskers encompassed 1.5 times the interquartile range. The differences between every two groups were compared through the Kruskal-Wallis test (*p < 0.05; **p < 0.01; ***p < 0.001). **(E)** Differences in 20 signatures (immune-related signature, DNA repair-related signature, and stroma-related signature) between different clusters. The asterisks represented the statistical *p*-value (*p < 0.05; **p < 0.01; ***p < 0.001). **(F)** Alluvial diagram showed the association of molecular subtypes in different clusters.

By analyzing the GSVA enrichment, we were able to better understand the biological characteristics behind different subtypes. Figure 2F and Supplementary Table S6 showed that subtype B was enriched in cancer and stroma-related pathways, such as the cell adhesion molecule CAMs, ECM receptor interaction, and focal adhesion pathways. Considering the strong relationship between the angiogenesis subtype and the immune activity, we investigated the immune cell infiltration in both clusters using the CIBERSORT method (Supplementary Table S7). Figure 3A showed that activated CD4 memory T cells, CD8^+^ T cells activated NK cells, and activated dendritic cells tended to be higher in cluster A compared to cluster B, whereas neutrophils and various types of macrophage cells tended to be higher in subtype B. The ESTIMATE package was also used to evaluate the TME score of both subtypes (immune score, stromal score, and estimate score). There was a greater immune and stromal score for subtype B than for subtype A (Figure 3B). In addition, we developed a heatmap using ssGSEA to visualize and compare 23 immune infiltrating cell subpopulations in different subtypes (Figure 3C; Supplementary Table S8). Interestingly, subtype B had a high concentration of immune cells, including antitumor lymphocyte subsets such as activated CD8+/CD4+ T cells and NK T cells. Evidence suggested that tumors with immune-excluded phenotypes retain many immune cells in the stroma surrounding their nests rather than penetrating their parenchyma (Chen and Mellman, 2017). As a result, we hypothesized that an effective anti-tumor immune response is inhibited by the abundant stromal component in subtype B. Subsequently, the MCPcounter algorithm confirmed that fibroblasts and endothelial cells were heavily infiltrated in subtype B (Figure 3D; Supplementary Table S9). Subsequent analysis revealed that the stroma pathway was significantly activated in subtype B (Figure 3E), exhibited processes associated with EMT, Pan-F-TBRS (pan-fibroblast TGF-β response signature), and WNT-targeting pathways (Mariathasan et al., 2018). Researchers from Marisa et al. identified four major molecular subtypes in CC patients (CIN, dMMR, CSC, and KRASm). They found that dMMR tumors had immunological upregulation and cell growth, while CSC was associated with the downregulation of cell cycle pathways and poor prognosis (Marisa et al., 2013). Consistent with previous findings, dMMR and CSC subtypes were mainly concentrated in patients with subtype B Figure 3F. Our findings confirmed that the two subtypes have distinct immune infiltration characteristics.

### Identification of different subtypes of DEGs

**FIGURE 4:**
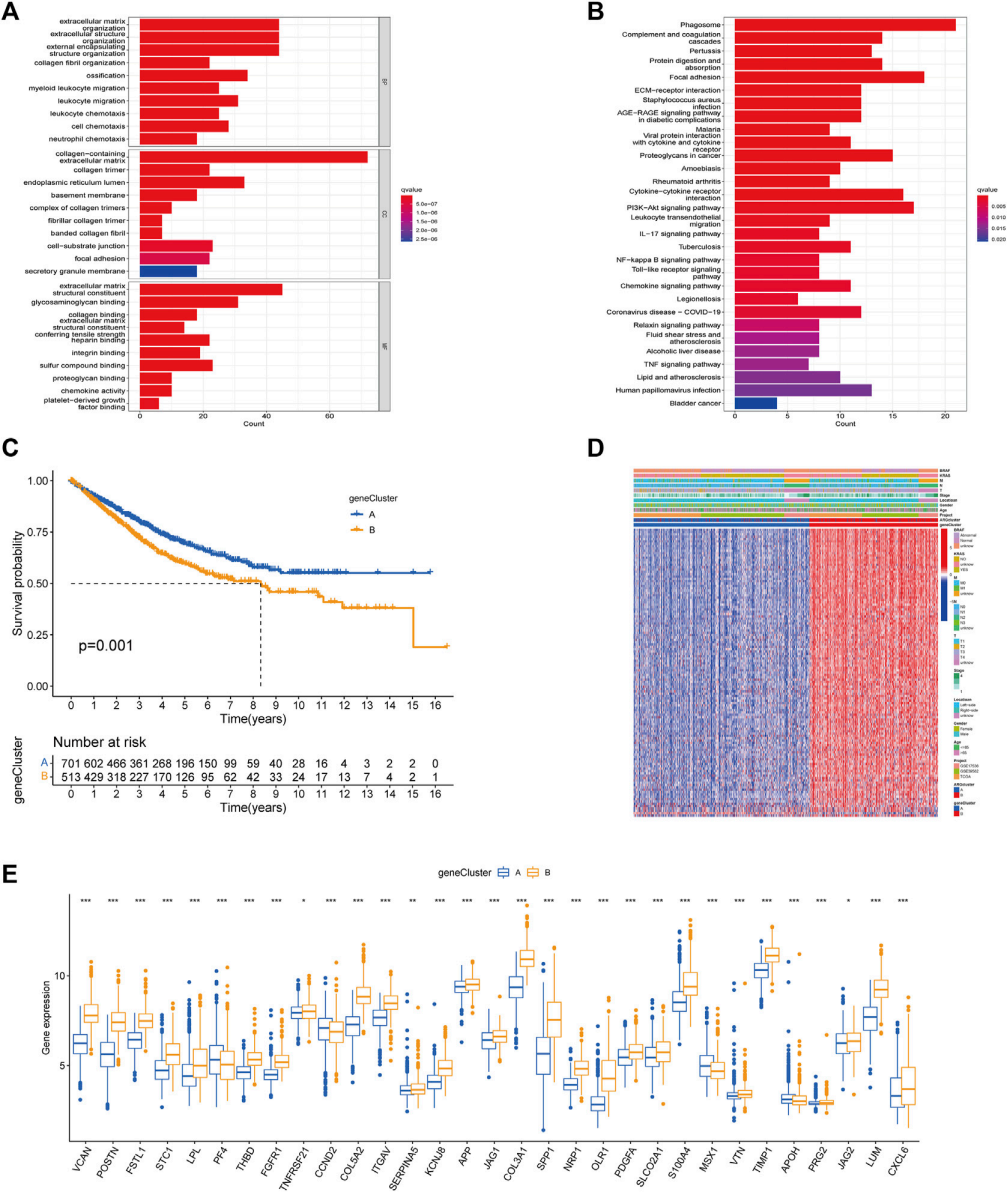
Based on DEGs to identify gene subtypes. **(A, B)** Enrichment analysis of DEGs between two subtypes using GO and KEGG. The color depth of the barplots represented the number of genes enriched. **(C)** K-M curves for OS of the two gene subtypes (Log-rank test, *p* = 0.001). **(D)** Relationship between the two gene subtypes and clinicopathological features. **(E)** Expression differences of 36 ARGs between the two gene subtypes. The asterisks represented the statistical *p*-value (*p < 0.05; **p < 0.01; ***p < 0.001).

To better understand the potential genetic alterations and expression perturbations in the phenotypic subtype, we used the LIMMA package to identify 254 DEGs between subtypes and performed a functional enrichment analysis (Figures 4A, B; Supplementary Table S10). DEGs enrichment results at GO showed biological processes associated with the extracellular matrix, and KEGG analysis showed pathways related to cancer and stroma. We identified 123 prognostic genes using univariate cox regression analysis based on 254 DEGs (*p* < 0.01) (Supplementary Table S11). To investigate the angiogenesis-associated transcriptional expression changes in both subtypes, we then performed an unsupervised consensus clustering analysis, yielded two distinct gene subtypes (Supplementary Figure S4). Log-rank test comparing OS in patients with A and B gene subtypes (Figure 4C) showed an advantage for patients with the gene subtype A (*p* = 0.001). Heatmap illustrated the correlation between gene subtypes and clinical characteristics of CRC patients (Figure 4D). Comparing the expression levels of the 36 ARGs in gene subtypes was shown in Figure 4E. Different gene subtypes showed significant differences in ARG expression, which was consistent with angiogenesis subtypes.

### Construction of ARGscore and validation

**FIGURE 5:**
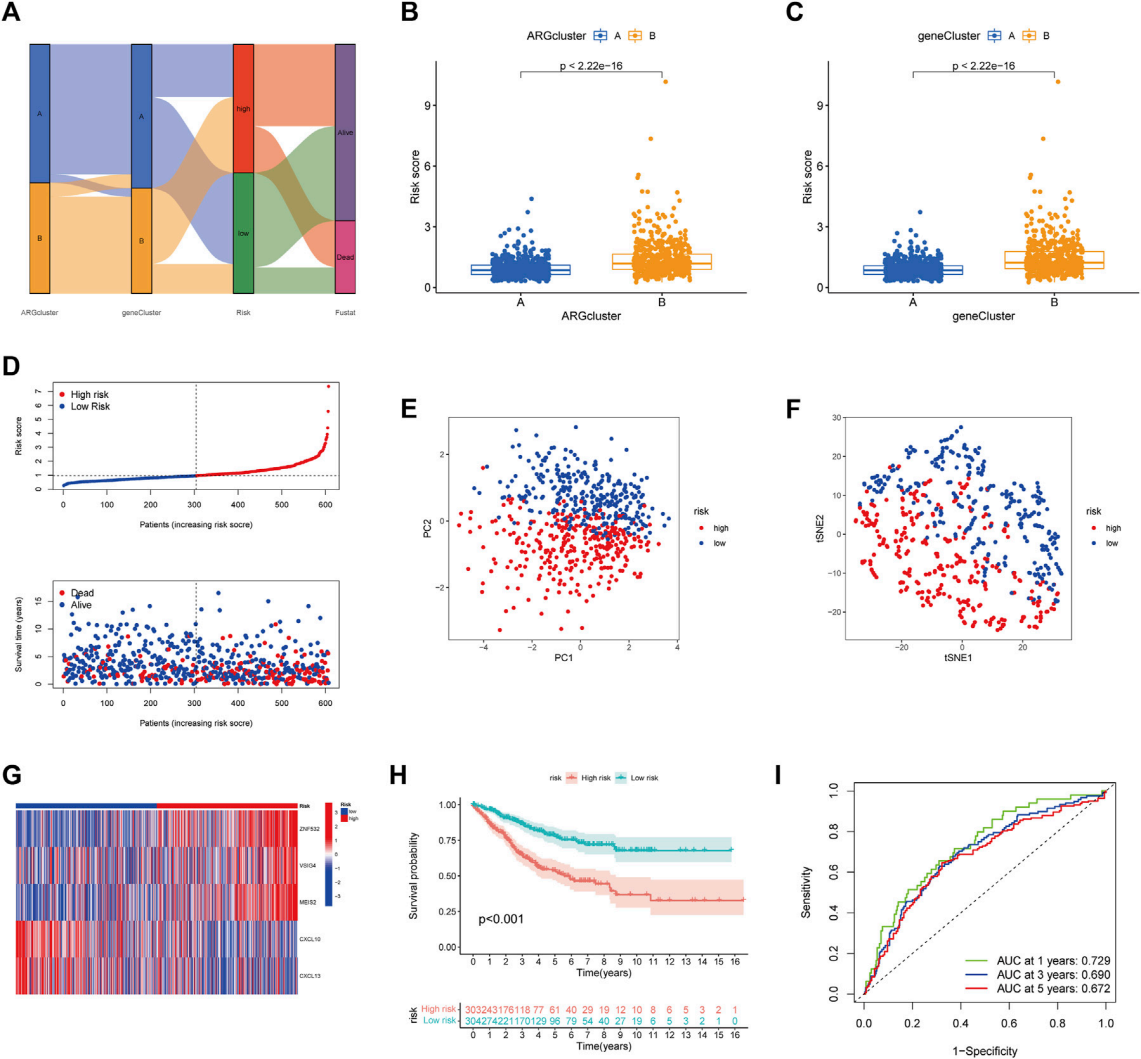
Construction of ARGscore in the train set. **(A)** Alluvial diagram of the subgroup distribution of different ARGscore and survival outcomes. **(B)** Differences in ARGscore between angiogenesis subtypes. **(C)** Differences in ARGscore between gene subtypes. **(D)** Ranked dot and scatter plots displayed the distribution of ARGscore and the survival status of patients. **(E, F)** The PCA and t-SNE analysis demonstrated that the patients in the different risk groups were distributed in two directions. **(G)** Expression heatmap of five prognostic genes of the model in high- and low- ARGscore groups. **(H)** K-M analysis of OS between the two subgroups (Log-rank, *p* < 0.001) **(I)** ROC curves for predicting survival and sensitivity at 1, 3, and 5 years based on ARGscore.

Considering the complexity and individual heterogeneity of angiogenesis, the ARGscore was developed to quantify the pattern of angiogenesis in CRC patients. The distribution of patients across two angiogenesis subtypes, two gene subtypes, and two ARGscore subgroups was shown in Figure 5A. We randomly assigned patients in a 1:1 ratio to train (*n* = 607) and test (*n* = 607) sets. Lasso and Multivariate Cox analysis were performed for prognostic genes to select the most accurate prognostic indicators further (Supplementary Figure S5). According to the minimum partial likelihood deviation, 11 relevant genes remained. Based on Akaike information criterion (AIC) values, we then eventually acquired five genes (VSIG4, CXCL10, CXCL13, MEIS2, ZNF532). The ARGscore calculated by the formula = (0.2754) *expression level of ZNF532+(0.1833) * expression level of VSIG4+(0.1599) * expression level of MEIS2+(−0.1619) * expression level of CXCL10+(−0.1215) * expression level of CXCL13. We found significant differences between angiogenesis and gene subtypes, with patients of subtype B having higher ARGscore, suggested that ARGscore-high might be associated with stromal activation characteristics (Figures 5B, C). To categorize patients, the median ARG score was used. The distribution plot of risk of ARGscore revealed that the survival time decreased and mortality increased with increasing ARGscore (Figure 5D). PCA and t-SNE showed significant dimensionality between the distinct subgroups (Figures 5E, F). The heatmap of model prognostic gene expression in various subgroups was also shown in Figure 5G. To determine the prognostic power of ARGscore in predicting survival outcomes, Kaplan-Meier survival curves showed a significantly longer OS for patients with ARGscore-low (*p* < 0.001; Figure 5H). The AUC values of 0.729, 0.690, and 0.672 were also used to determine ARGscore survival rates at 1, 3, and 5 years (Figure 5I). Next, we aimed to evaluate the predictive performance of the ARGscore for the test and all sets (Supplementary Figures S6, S7). According to the formula used for the training cohort, patients were also assigned to distinct subgroups. Similarly, survival analysis showed that ARGscore-low patients had a higher OS than ARGscore-high patients. It was found that ARGscore still had a good AUC value for predicting 1-, 3-, and 5-year survival probabilities, which suggested that ARGscore is a good maker for assessing CRC prognosis.

### Prognostic model validation of five ARGs expression levels

**FIGURE 6:**
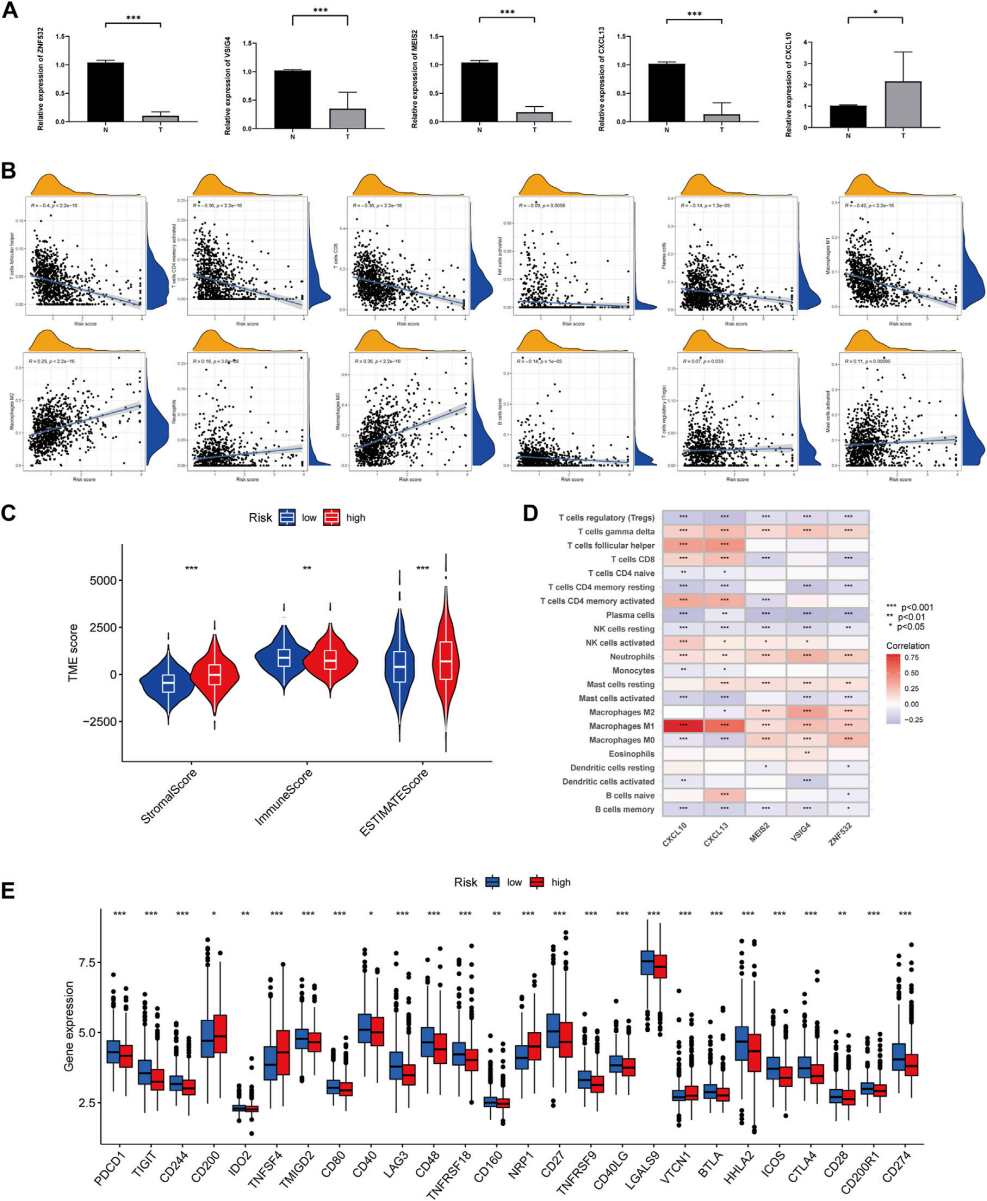
Comparison of TME and immune checkpoints between two subgroups. **(A)** Expression levels of five angiogenesis-related genes of prognostic signature in colorectal cancer tissues and corresponding normal tissues by RT-qPCR. The asterisks represented the statistical *p*-value (*p < 0.05; ***p < 0.0001). **(B)** Correlation between immune cell type and ARGscore. The asterisks represented the statistical *p*-value (*p < 0.05; **p < 0.01; ***p < 0.001). **(C)** Correlation between ARGscore and immune and stromal score. The asterisks represented the statistical *p*-value (*p < 0.05; **p < 0.01; ***p < 0.001). **(D)** Correlation between the abundance of immune cells and five genes in the prognostic model. **(E)** Differences in expression of immune checkpoints in high and low-risk groups. The asterisks represented the statistical *p*-value (*p < 0.05; **p < 0.01; ***p < 0.001).

RT-qPCR was used to measure the expression levels of five prognostic genes in eight CRC tissues and normal adjacent tissues. As shown in Figure 6A, the expression levels of VSIG4, ZNF532, MEIS2, and CXCL13 were downregulated, while CXCL10 was elevated between colorectal cancer and normal tissues.

### Exploration of the clinical relevance of ARGscore

To investigate the association between ARGscore and clinical characteristics, we examined the correlation between ARGscore and age, gender, stage, tumor location, BRAF mutation, and KRAS mutation (Supplementary Figure S8). To assess whether ARGscore could independently predict the value of OS in CRC patients. Multivariate and univariate analyses were performed to determine the prognostic independence of the clinical factors. As shown in Supplementary Figure S9, the training set showed differences in age, stage, and ARGscore, and we observed consistent results in the test set and the all set. In addition, we divided patients into subgroups based on clinical characteristics to evaluate the predictive power of ARGscore, and survival was generally worse in ARGscore-high patients compared with ARGscore-low patients (Supplementary Figure S10). The stratified analysis showed that ARGscore retained its predictive ability in different subgroups as well.

### Differences in TME across risk subgroups

CIBERSORT was used to assess the association between ARGscore and immune cell infiltration. On the scatter plot, ARGscore correlated positively with M0 and M2 macrophages, neutrophils, activated mast cells, and Tregs. Negative correlations were found with M1 macrophages, activated CD4 memory T cells, naive B cells, CD8 T cells, follicular helper T cells, activated NK cells, and plasma cells (Figure 6B). ARGscore-high was linked to a higher stromal score, while ARGscore-low was correlated with a higher immune score (Figure 6C). The abundance of immune cells was also evaluated in relation to five genes in the model. These five genes were significantly associated with immune cells (Figure 6D). Furthermore, we researched the relationship between the ARGscore and immune checkpoints. PD-L1, PD-1, and CTLA-4 were among 26 immune checkpoints that were differentially expressed between the two subgroups, as shown in Figure 6E.

### Association of ARGscore with CSC index, MSI, mutation, and drug sensitivity

**FIGURE 7:**
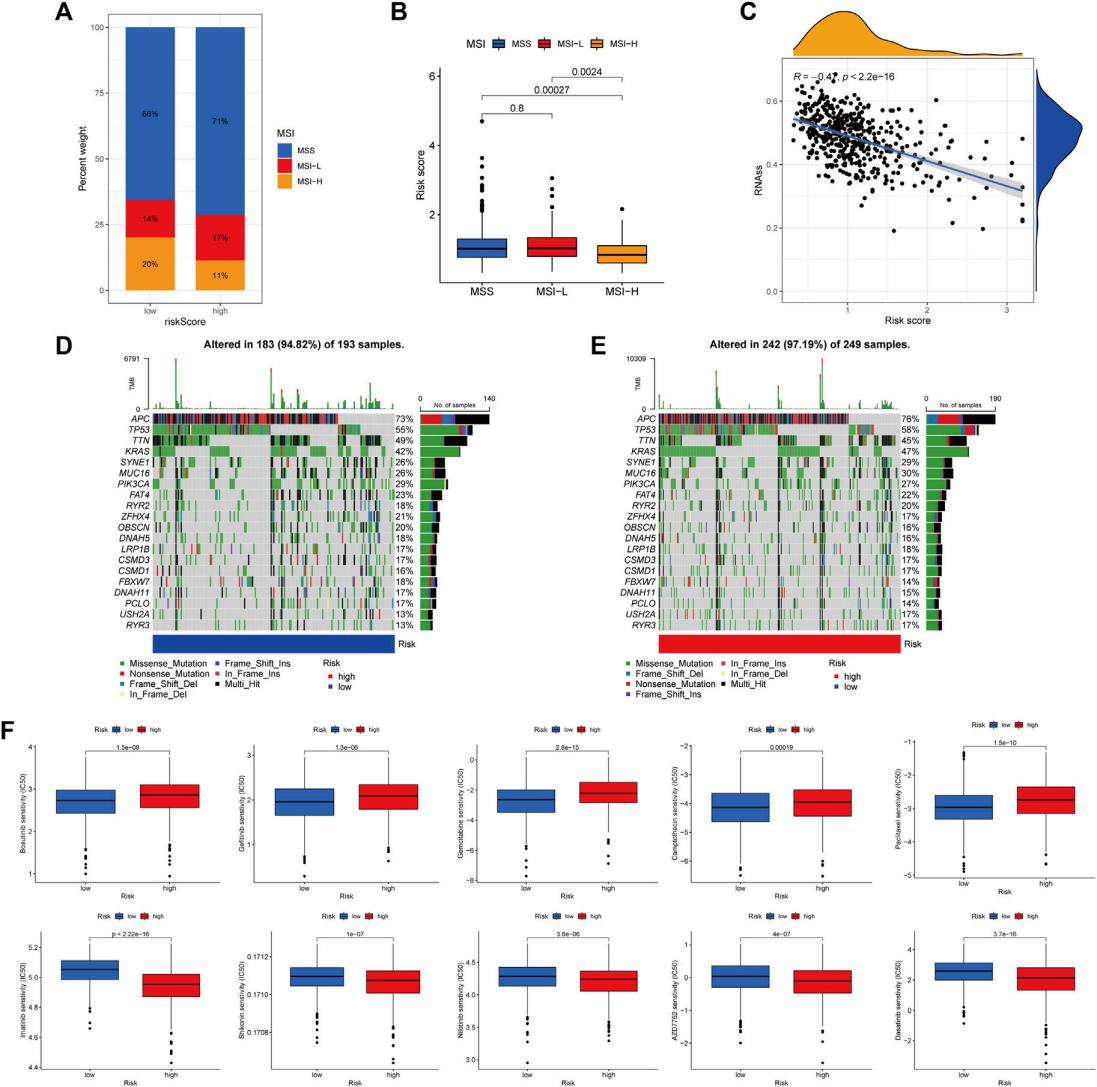
Comprehensive analysis of ARGscore in colorectal cancer. **(A, B)** Relationship between ARGscore and MSI. **(C)** Correlation between ARGscore and CSC index. **(D, E)** The waterfall plot of somatic mutation frequency was established on high and low ARGscore. The upper barplot showed TMB. The number on the right indicated the mutation frequency in each gene. The right barplot showed the proportion of each variant type. **(F)** Drug sensitivity analysis of ARGscore and chemotherapeutic drugs.

There is growing evidence that patients with MSI-H are more susceptible to immunotherapy and could benefit from immunotherapy. In the correlation analysis, ARGscore-low was associated with MSI-H, whereas ARGscore-high was associated with MSS (Figures 7A, B). In addition, ARGscore and CSC index values were combined to examine the correlation. The linear correlation between ARGscore and CSC index was displayed in Figure 7C. ARGscore was negatively correlated with the CSC index (*R* = −0.47, *p* < 2.2e-16), which indicated that cells with lower ARGscore had more pronounced stem cell characteristics and a lesser degree of differentiation. In the TCGA-COAD/READ cohort, we analyzed the differences in somatic mutation distribution between the two ARGscore subgroups. The two groups’ mutated genes with more than 25% mutation frequency were APC, TP53, TTN, KRAS, SYNE1, MUC16, and PIK3CA (Figures 7D, E). The ARGscore-high subgroup had a higher mutation frequency than the ARGscore-low subgroup. In contrast, mutation levels of TTN and PIK3CA were the exact opposite. Colorectal cancer chemotherapeutic agents were selected to assess sensitivity to chemotherapy in the distinct subgroups. Notably, patients with ARGscore-low have lower IC_50_ values for paclitaxel, bosutinib, gefitinib, gemcitabine, and camptothecin. In contrast, the IC_50_ values for the chemotherapeutic agents dasatinib, AZD7762, nilotinib, shikonin, and imatinib were significantly lower in patients with ARGscore-high. In conclusion, the results indicated that ARGs affected drug sensitivity (Figure 7F).

### Development and evaluation of a nomogram for predicting survival

**FIGURE 8:**
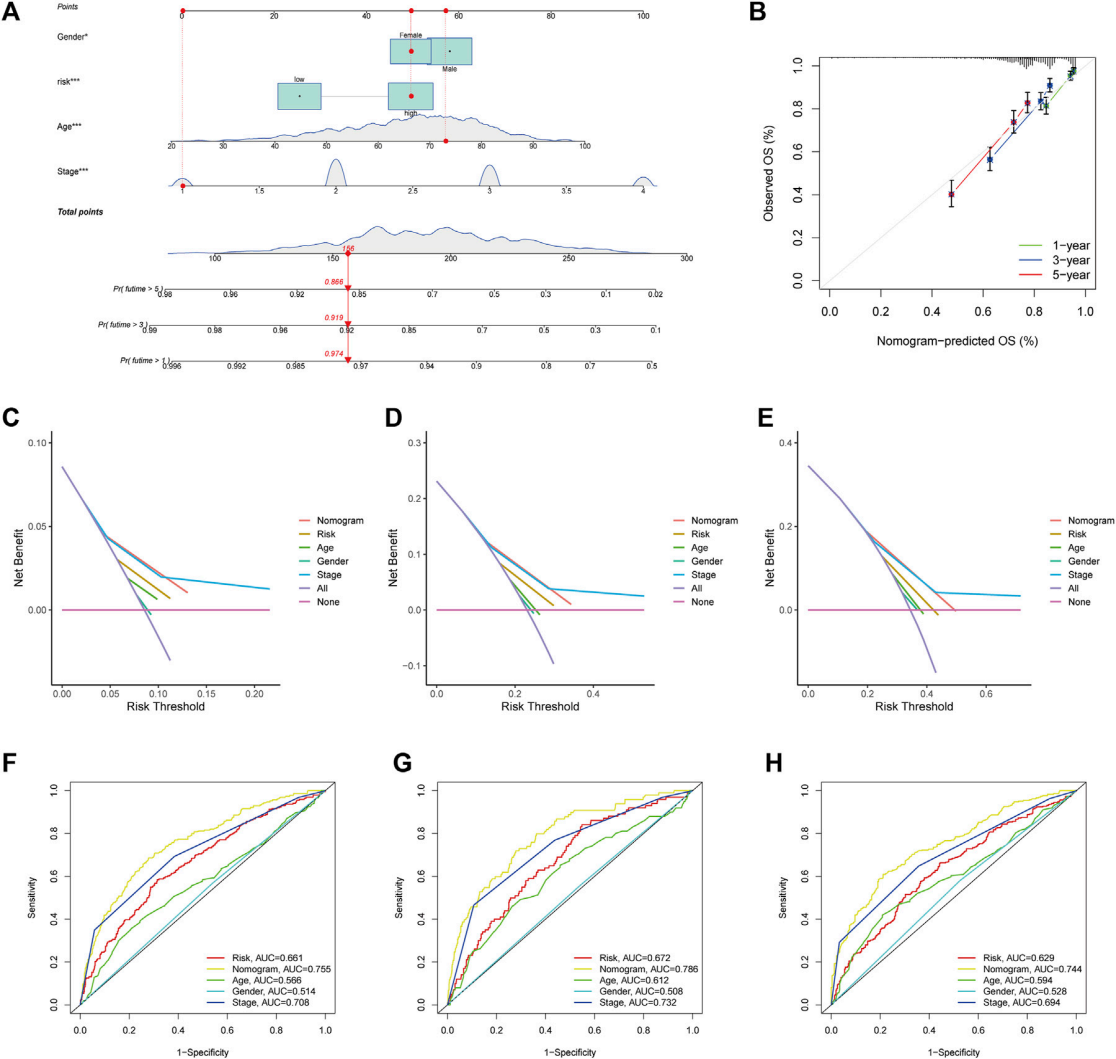
Development and validation of the nomogram. **(A)** Nomogram for predicting 1-, 3-, and 5-year OS in the entire cohort of colorectal cancer patients. **(B)** The Calibration curve of the nomogram. **(C–E)** The DCA curves of the nomograms compared for 1-, 3-, and 5-year OS in CRC, respectively. **(F–H)** ROC curves comparing 1, 3, and 5 years in patients with colorectal cancer.

Considering the high correlation between ARGscore and patient prognosis, to quantify the individual risk assessment of CRC patients, we incorporated clinical parameters to build the Nomogram. This Nomogram included ARGscore and patient gender, age and stage, and was used to estimate overall patient survival at 1, 3 and 5 years (Figure 8A). The calibration curve showed the high accuracy between the actual observed and predicted parameters (Figure 8B). In addition, this prognostic model including different clinical factors showed a better advantage in predicting survival (Figures 8C–E). The nomogram was also compared to other clinical variables for predictive accuracy. Figures 8F–H showed that the AUC values for Nomogram at 1, 3 and 5 years were 0.786, 0.755 and 0.744, respectively, indicated a better predictive ability for survival.

## Discussion

CRC is difficult to be detected in its early stages, and patients who present with clinical symptoms and signs are often already in advanced stages with poor prognoses. Compared with conventional chemotherapy and targeted therapy, immunotherapy has changed the outlook of CRC treatment due to its outstanding effectiveness. Colorectal cancer tumorigenesis and progression are significantly influenced by TME, and tumor metastasis is largely determined by angiogenesis (Hinshaw and Shevde, 2019). Most studies to date have focused solely on a single angiogenesis-related gene. Therefore, the combined effects of multiple ARGs and the immune microenvironment have not been adequately characterized. This study revealed transcriptional and genetic changes in ARGs in colorectal cancer. Two distinct subtypes were identified based on the expression of 36 ARGs. Subtype B patients had worse overall survival compared with subtype A patients. Furthermore, the two subtypes differed significantly in their TME characteristics. We developed and validated a robust and valid prognostic predictor, ARGscore. Immune activation and immune suppression were associated with lower and higher ARGscore, respectively. The clinicopathological features, prognosis, mutations, TME, MSI, immune checkpoints, CSC index, and drug sensitivity of patients with low and high ARGscore differed significantly. Lastly, a quantitative nomogram was developed by combining ARGscore with clinical characteristics to improve its performance and facilitate its use. By using this prognostic model, colorectal cancer patients could be stratified for prognosis, which will contribute to a better understanding of the molecular mechanisms of cancer.

TME influenced colorectal cancer tumorigenesis and progression. Immune cells such as tumor-infiltrating T cells (CD4+/CD8+ T cells), M1 macrophages, and NK cells have been shown to be associated with immune responses (Topalian et al., 2016; Hinshaw and Shevde, 2019; Zeng et al., 2020). A good prognosis is associated with the density of infiltrating T cells in CRC tissues [(Governa et al., 2017; Kuwahara et al., 2019)]. Tfh (follicular helper T cell) is important for an effective antitumor response, and Tfh also promotes intra-tumor B-cell differentiation, thereby improving antitumor immunity (Gu-Trantien et al., 2013). In our result, there was a better prognosis for patients with ARGscore-low and subtype A, with higher infiltration of activated CD4^+^, CD8^+^ T cells, and Tfh, suggesting a potential role of ARG in the development of colorectal cancer.

Even though immune cells were abundant in the immune-excluded phenotype, they remained in the stroma around tumor cells rather than permeating them. In some cases, the stroma is contained within the tumor envelope, while in others, it penetrates the tumor itself, making it appear as though the immune cells are inside the tumor [(Gajewski, 2015; Joyce and Fearon, 2015)]. Consistent with the above definition, in our study, subtype B showed a highly enhanced stroma activation state, including high expression of TGF-β pathways, angiogenesis, and WNT signaling pathways, which are considered T cell suppressive. Our immunophenotypic classification of the different angiogenic patterns is confirmed by the cell-permeable properties of TME in subtype B. As a result, it is not surprising that subtype B has an activated immune function but poor prognoses.

It has been shown that B cells infiltrating metastatic colorectal cancer were associated with a good prognosis, and B cell infiltrating patients have a significantly lower recurrence rate (Berntsson et al., 2016). Therefore, B cells might play a role in anti-tumor immunity and provide a new target for colorectal cancer immunotherapy. Our study found that naive B cells were significantly more prevalent in the subtype A and ARGscore-low subgroups. This suggested that B Cell infiltration might have inhibited tumor growth in CRC.

Cancer immunotherapy has emerged as a new therapeutic option with tumor immunology and molecular biology development. Immunotherapies include immune checkpoint inhibitor (ICI), cellular therapies, and therapeutic antibodies. ICI therapies have shown considerable survival advantages over conventional therapies [(Rotte, 2019; Marin-Acevedo et al., 2021)]. There was a positive correlation between MSI status and better survival in CRC compared to MSS [(Ganesh et al., 2019)]. We found higher expression levels of PD-1, PD-L1, and CTLA4 in the ARGscore-low subgroup. Our study observed that the ARGscore-low subgroup exhibited high infiltration of multiple immune cells and high expression of PD-1 and MSI-H states. The results suggested that patients with low ARGscore might respond better to immune checkpoint blockade.

Limitations of this study remain. All samples used in this study, derived from the public databases, were retrospective. This may have introduced a selection bias into the analysis. In addition, additional, important clinical variables were not available and analyzed in the dataset species. It is, therefore, necessary to confirm our findings with large prospective cohort studies as well as *in vitro* and *in vivo* experiments.

## Data Availability

The original contributions presented in the study are included in the article/Supplementary Material, further inquiries can be directed to the corresponding author.
