# Supplementary material for: Identification of angiogenesis-related subtypes, the development of prognostic models, and the landscape of tumor microenvironment infiltration in colorectal cancer
Source: Front Pharmacol. 2023 Feb 22;14:1103547. doi: 10.3389/fphar.2023.1103547 (PMC9992542; doi:10.3389/fphar.2023.1103547)
Supplement: Supplementary file 2 [file Table1.DOCX]

**Supplementary Figures**


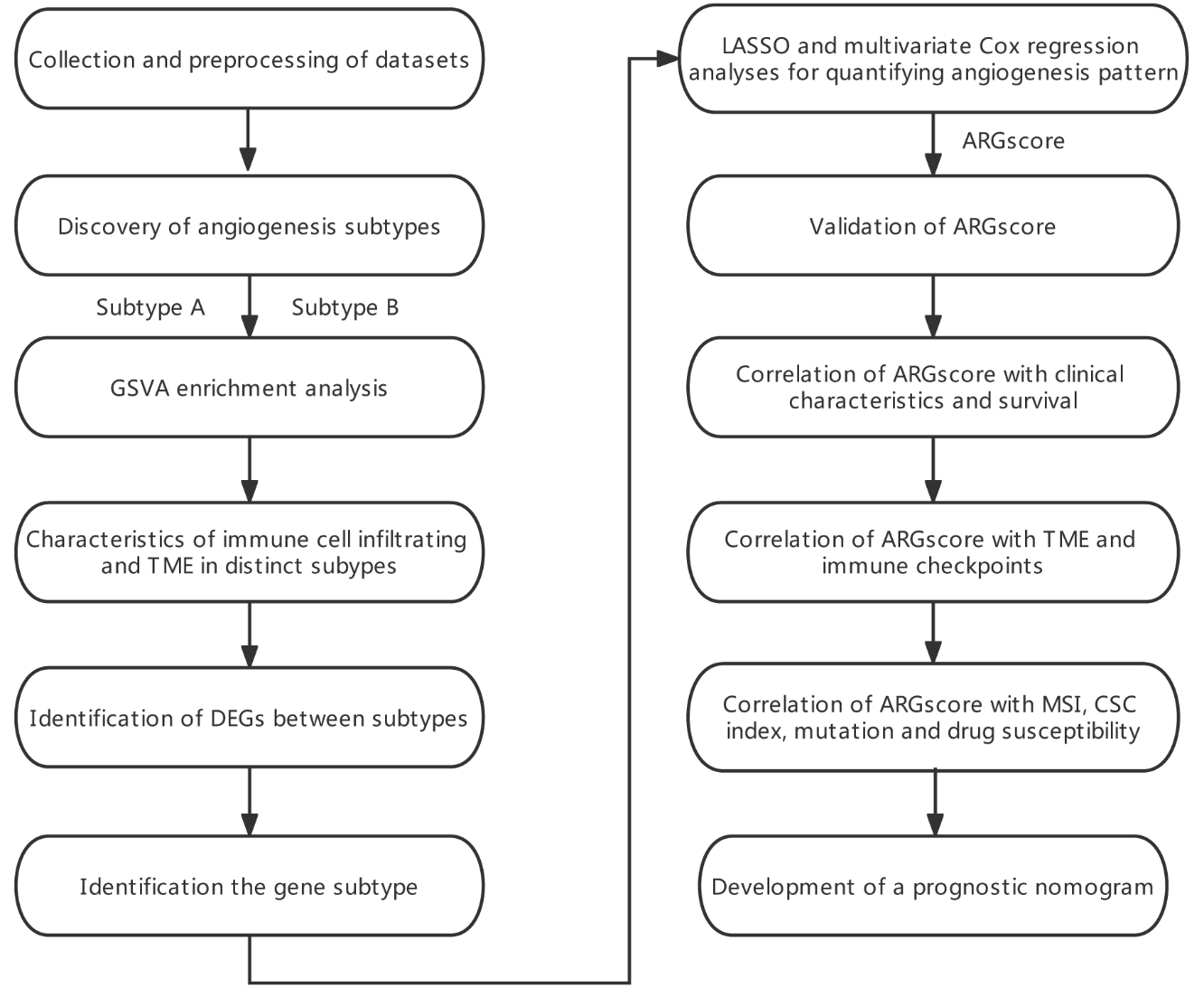


**Figure S1** The whole analytical process of the study.


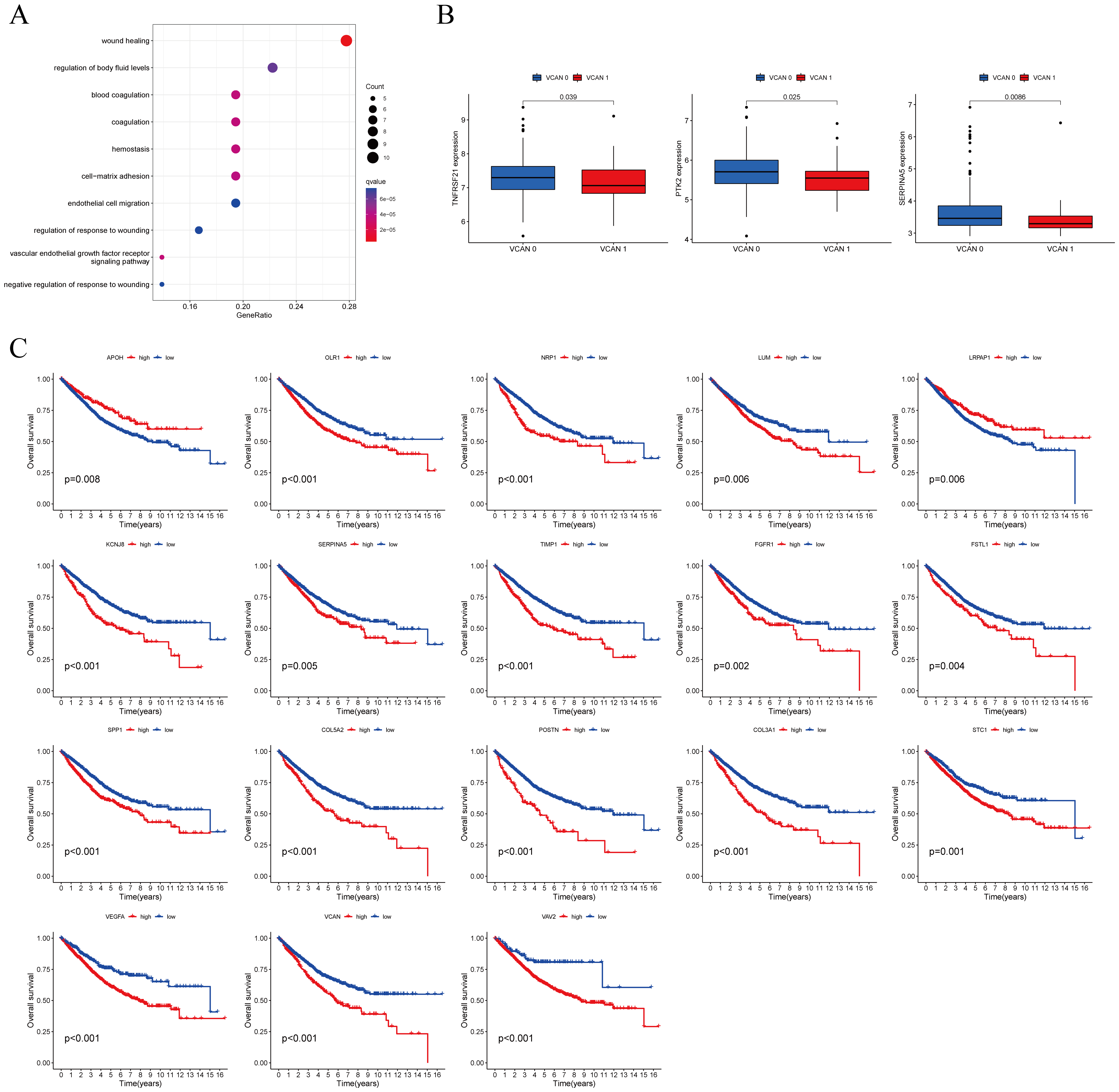


**Figure S2** (A) GO enrichment analysis of the 36 angiogenesis-related genes. (B) The relationship between VACN mutation and expression level of ARGs in CRC. 0, wild; 1 Mutation. (C) The K-M curves of prognosis-related ARGs.


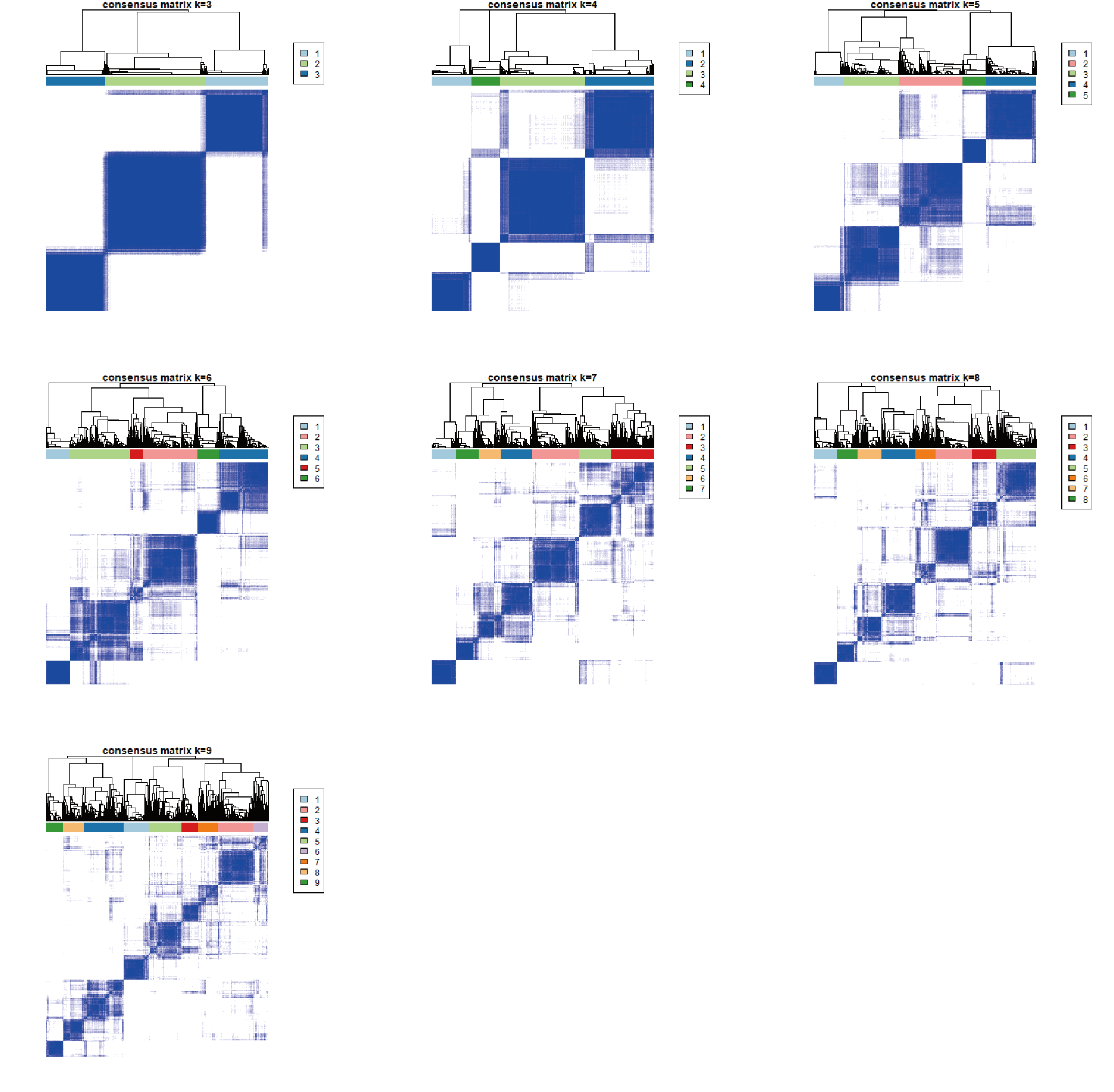


**Figure S3** Unsupervised clustering of angiogenesis-related genes and Consensus matrix heatmaps for k = 3-9.


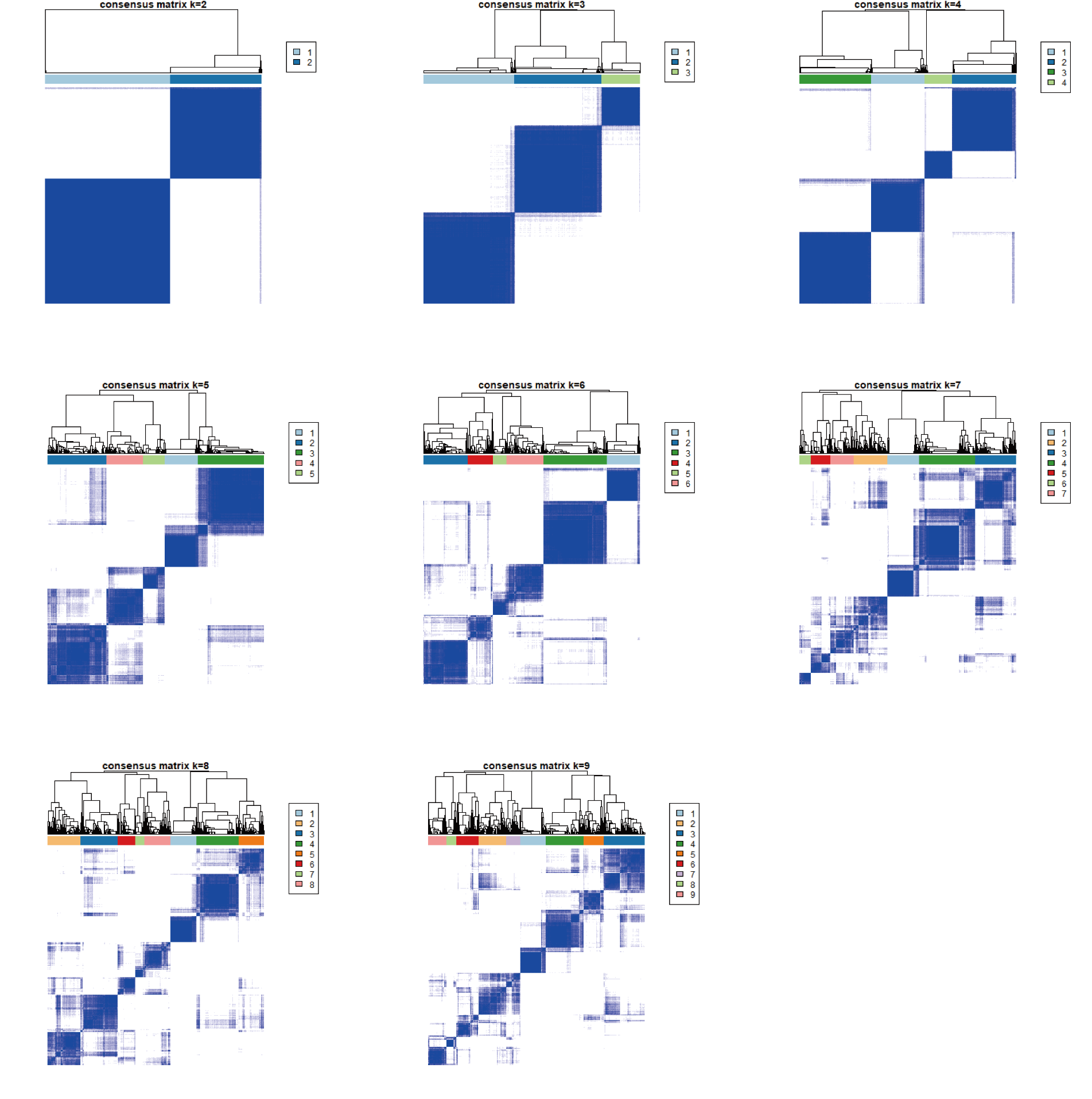


**Figure S4** Identification of gene subtypes based on DEGs among two subtypes in CRC cohort.


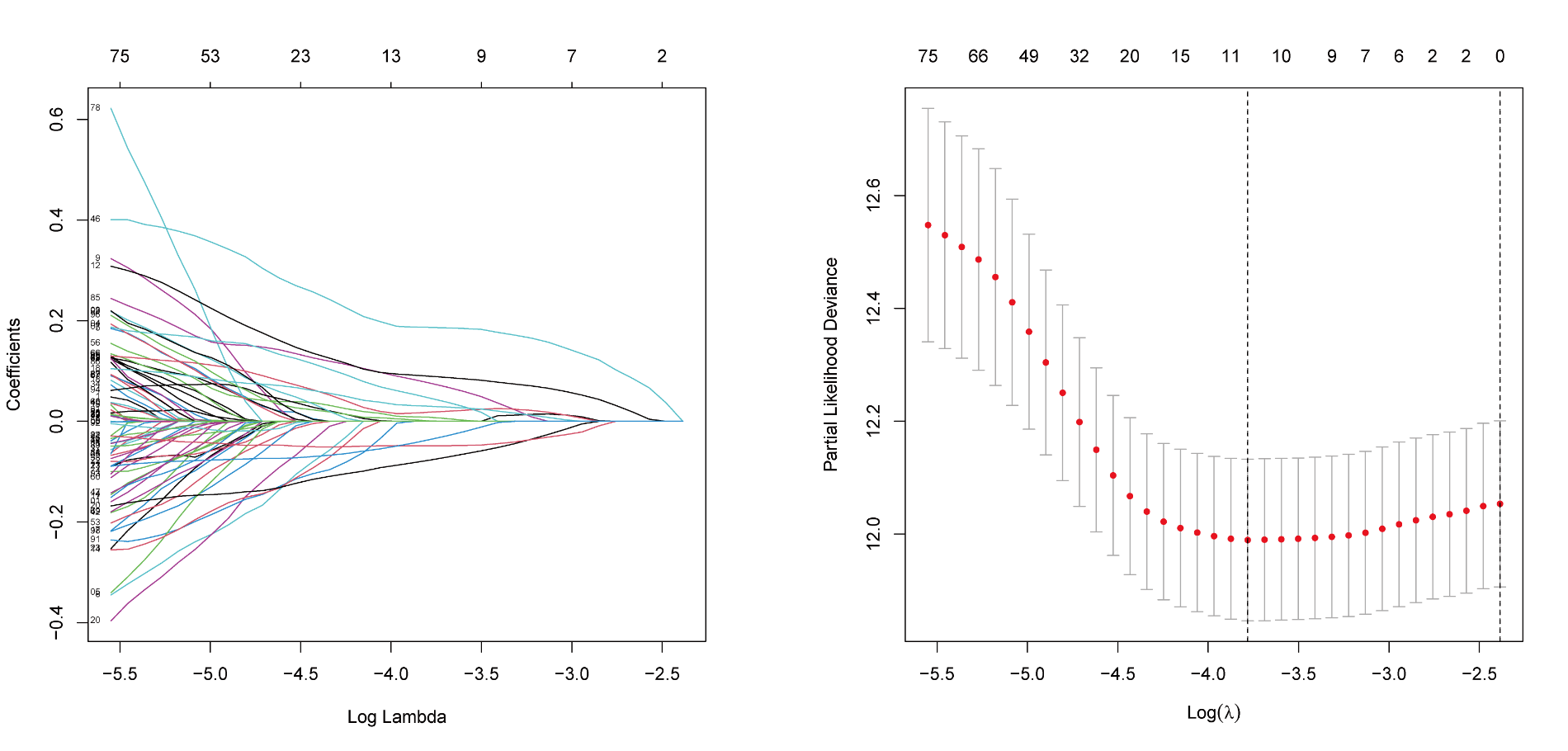


**Figure S5** The LASSO regression analysis and partial likelihood deviance on the prognostic genes.


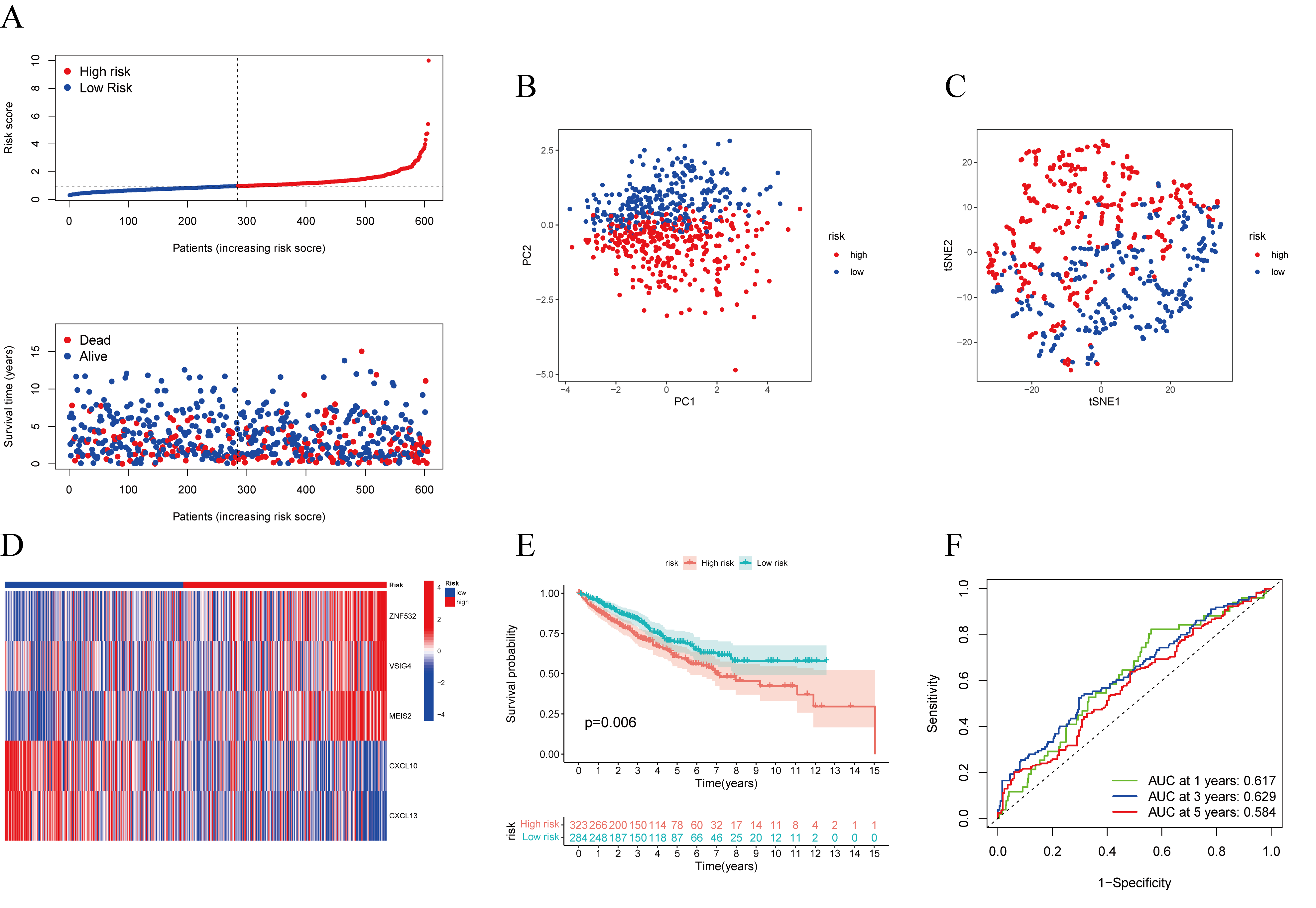


**Figure S6** Validation of ARGscore in the test set. (A) The ranked dot plot indicates the ARGscore distribution and scatter plot presenting the patients’ survival status. (B-C) The PCA and t-SNE analysis demonstrated that the patients in the different risk groups were distributed in two directions. (D)Expression heatmap of 5 prognostic genes of the model in high- and low- ARGscore groups. (E) K-M analysis of the OS between the two subgroups. (F) ROC curves to predict the sensitivity and specificity of 1-, 3-, and 5-year survival according to the ARGscore.


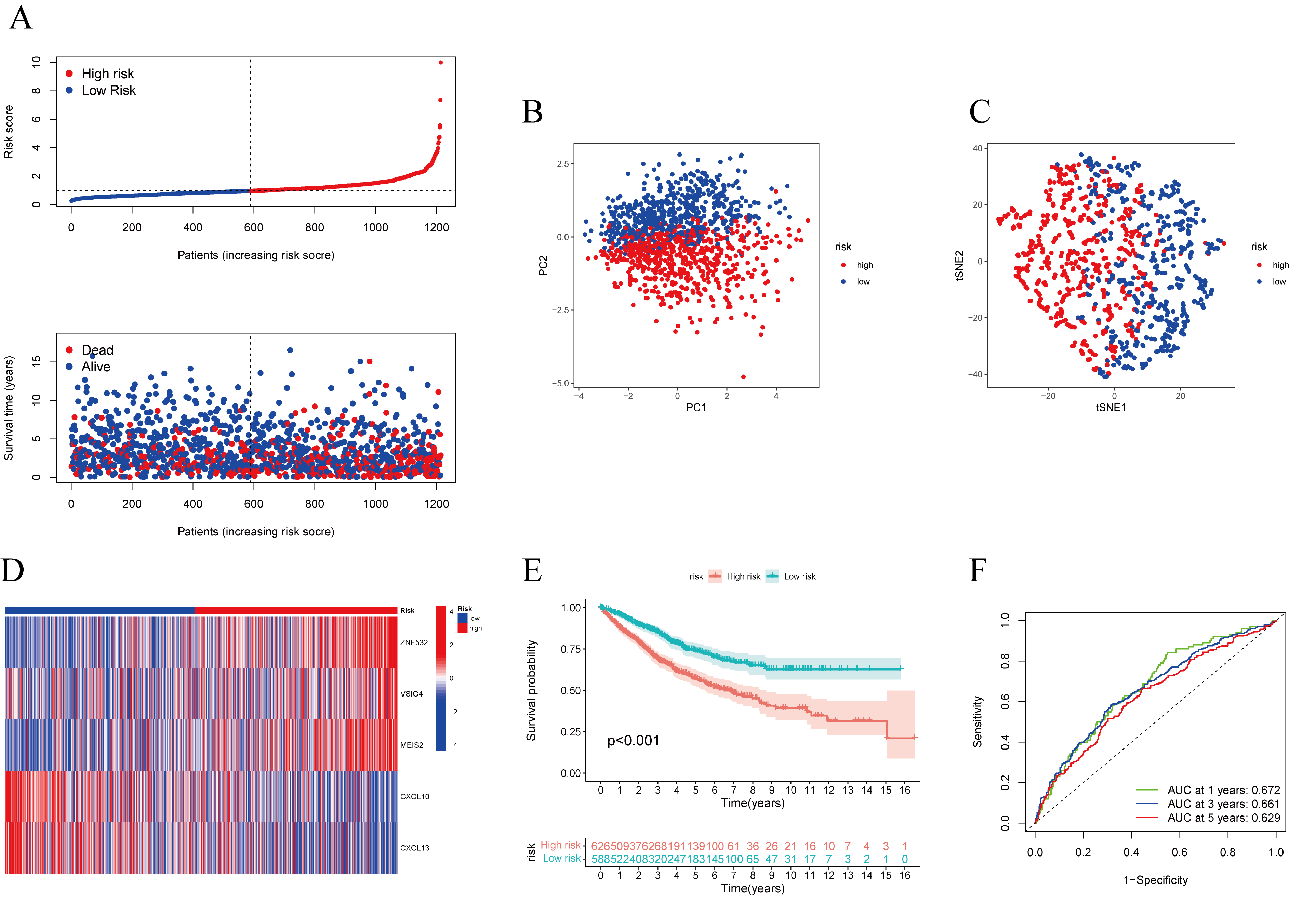


**Figure S7** Validation of ARGscore in the all set. (A) The ranked dot plot indicates the ARGscore distribution and scatter plot presenting the patients’ survival status. (B-C) The PCA and t-SNE analysis demonstrated that the patients in the different risk groups were distributed in two directions. (D)Expression heatmap of 5 prognostic genes of the model in high- and low- ARGscore groups. (E) K-M analysis of the OS between the two subgroups. (F) ROC curves to predict the sensitivity and specificity of 1-, 3-, and 5-year survival according to the ARGscore.


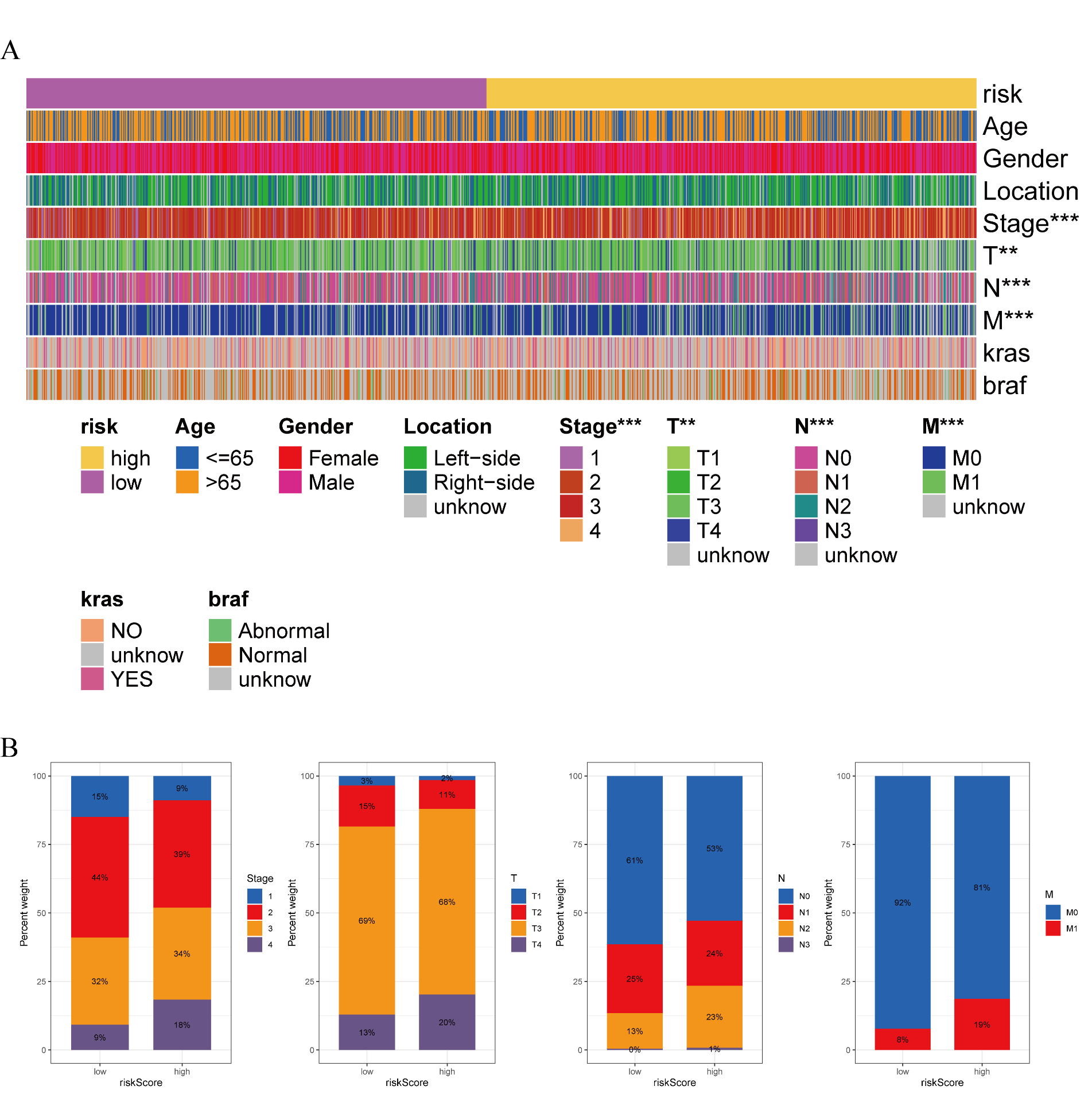


**Figure S8** The correlation between the ARGscore and clinical features. (A) The Correlation between ARGscore and age, gender, tumor location, stage, TNM stage, BRAF mutation, and KRAS mutation. (B) The bar chart indicates the percentage of staging, T, N, and M.


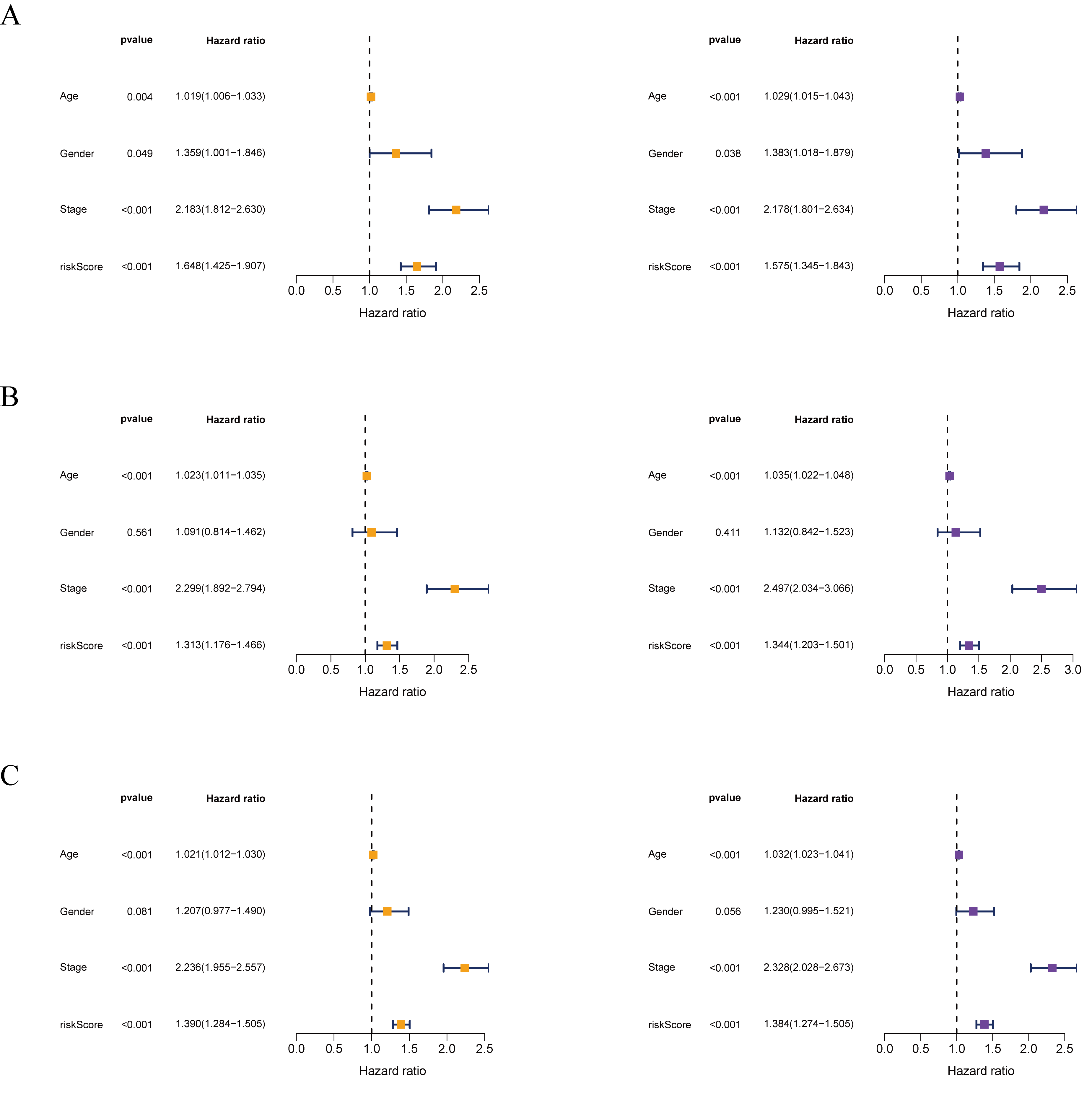


**Figure S9** The correlation and independent prognosis analysis of ARGscore and clinicopathological variables. (A) Univariate and multivariate analyses showed the prognostic value of the ARGscore in the train set. (B) Univariate and multivariate analyses showed the prognostic value of the ARGscore in the test set. (C) Univariate and multivariate analyses showed the prognostic value of the ARGscore in the all set.


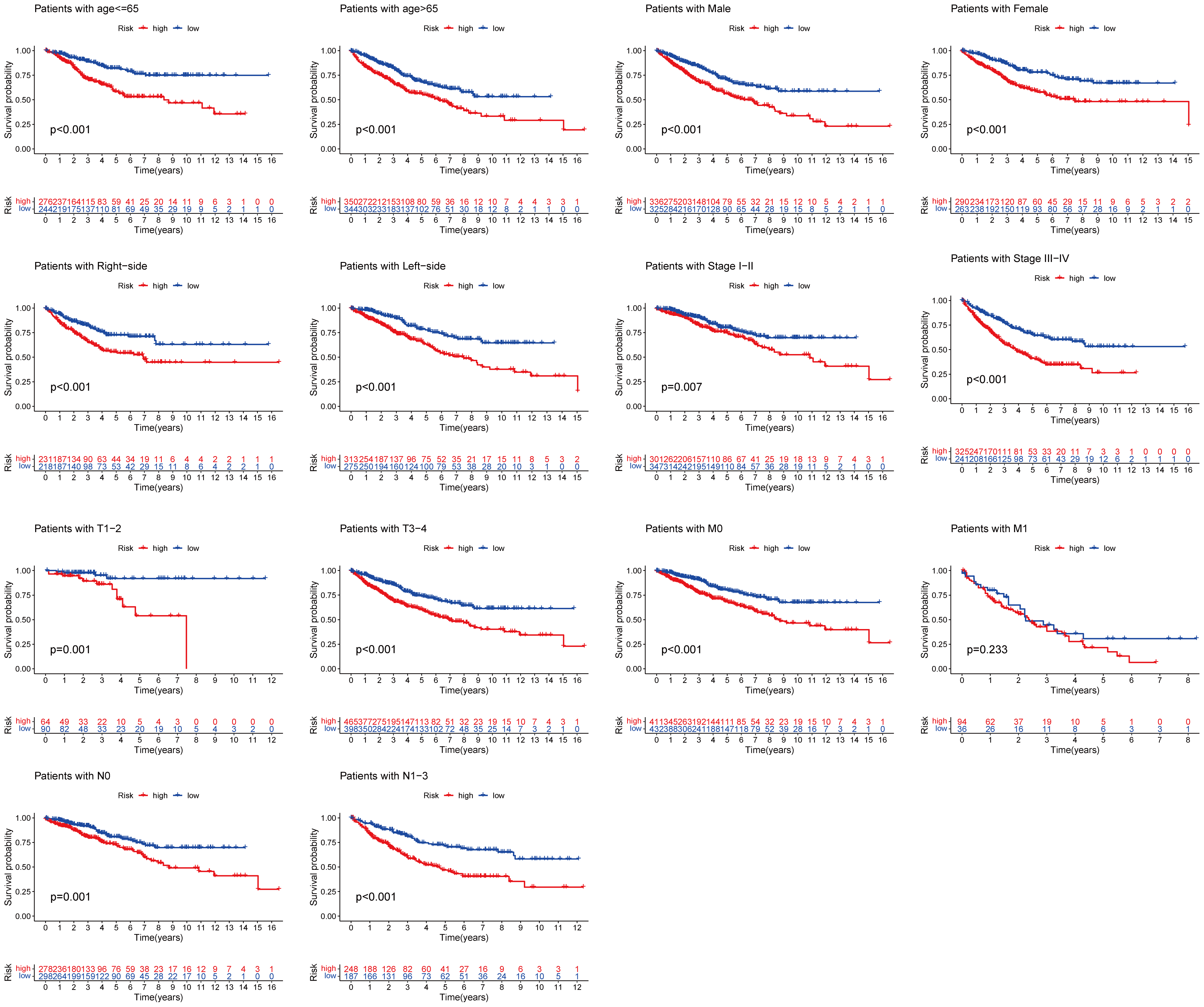


**Figure S10** Stratification analysis of the ARGscore in CRC. Age (age ≤ 65 and age > 65 years old); Gender (female and male); Tumor location (left-side or right-side); Tumor stage (I-II or III-IV); T (T1-2 or T3-4); M (M0 and ​M1); N (N0 or N1-N3)
